# Effect and Mechanism of siRNAs Targeting IL-1β/TNF-α Combined with BMSCs Transplantation in Ameliorating Rheumatoid Arthritis in Rats

**DOI:** 10.3390/vetsci9100531

**Published:** 2022-09-28

**Authors:** Shifeng Pan, Lijun Wang, Bingxing Wu, Hua Xing

**Affiliations:** 1 College of Veterinary Medicine, Yangzhou University, Yangzhou 225009, China; 2 Jiangsu Co-Innovation Center for Prevention and Control of Important Animal Infectious Diseases and Zoonoses, Yangzhou 225009, China

**Keywords:** siRNAs targeting IL-1β/TNF-α, bone marrow mesenchymal stem cells, rheumatoid arthritis, NF-κB signaling pathway, rats

## Abstract

**Simple Summary:**

The study aimed to explore the effect and potential mechanisms of siRNAs targeting IL-1β/TNF-α combined with BMSCs transplantation in ameliorating RA in rats. Collagen-induced arthritis model rats were randomly treated with IL-1β/TNF-α siRNA, BMSCs and IL-1β/TNF-α siRNA + BMSCs for 28 days. Compared with PBS group, BMSCs, siRNA, siRNA + BMSCs treatment groups showed significant lower toe swelling value, the immobility time, the spleen index, serum contents of IL-1β and TNF-α. In addition, the DR-X results showed that the knee carton surface tended to smoothing without bone hyperplasia, suggesting that these three treatments were all able to successfully ameliorate RA symptoms. In addition, compared with PBS group, the protein expression of p-NF-κB-p65 was significantly reduced in the knee of siRNA + BMSCs rats. BMSCs labeled with BrdU were also found in the knee of rats. Moreover, the mRNA expression of IL-1β, TNF-α and NF-κB-P65 in spleen tissue of siRNA + BMSCs rats were all significantly inhibited. Our results demonstrated that IL-1β/TNF-α siRNA, BMSCs and IL-1β/TNF-α siRNA + BMSCs were able to ameliorate RA inflammation by inhibiting the activation of NF-κB signaling pathways and reducing the erosion of articular cartilage, and IL-1β/TNF-α siRNA + BMSCs treatment showed synergism effects. Our study provides a new idea for gene and stem cell therapy for RA.

**Abstract:**

Background: Rheumatoid arthritis (RA) is an autoimmune disease. Bone marrow mesenchymal stem cells (BMSCs) have multilineage differentiation and anti-inflammatory potential, and small interfering RNAs (siRNAs) can inhibit the target gene expression, which make them suitable for ameliorating RA. The current study was aimed to explore the effect and potential mechanisms of siRNAs targeting IL-1β/TNF-α combined with BMSCs transplantation in ameliorating RA in rats. Methods: Collagen-induced arthritis (CIA) model rats were randomly divided into five groups: PBS (Model control group), methotrexate (Positive drug treatment group), BMSCs (BMSCs transplantation group), siRNA (IL-1β/TNF-α siRNAs injection group), siRNA + BMSCs (Both IL-1β/TNF-α siRNAs injection and BMSCs transplantation group). After treatment for 0, 7, 14, 21, 28 days, the ameliorating effect was comprehensively assessed through results of the body weight, toe swelling value, the immobility time of forced swimming, the serum concentrations of IL-1β and TNF-α, knee joint DR-X imaging and pathological analysis as well as of IL-1β, TNF-α and NF-κB mRNA expression in spleen tissue. Furthermore, the potential underlying mechanism involving the NF-κB signaling pathways was also explored. Results: Compared with the PBS group, BMSCs, siRNA, siRNA + BMSCs treatment groups showed significant lower toe swelling value, immobility time, spleen index, serum contents of IL-1β and TNF-α. In addition, the DR-X results showed that the knee carton surface tended to smoothing without bone hyperplasia, suggesting that these three treatments were all able to successfully ameliorate RA symptoms. In addition, compared with the PBS group, the protein expression of p-NF-κB-p65 was significantly reduced in the knees of siRNA + BMSCs rats. BMSCs labeled with BrdU were also found in the knees of rats. Moreover, the mRNA expression of IL-1β, TNF-α and NF-κB-P65 in spleen tissue of siRNA + BMSCs rats were all significantly inhibited. Conclusions: Our results demonstrated for the first time that siRNA + BMSCs was able to ameliorate RA inflammation by inhibiting the activation of NF-κB signaling pathways and reducing the erosion of articular cartilage, and siRNA + BMSCs treatment showed synergism effects in helping ameliorating the inflammation and cartilage repair of RA rats. Therefore, the results of our present study provide a new idea for gene and stem cell therapy for RA.

## 1. Background

Rheumatoid arthritis (RA) is a chronic autoimmune disease that is characterized by persistent synovitis, abnormal synovial hyperplasia, increased angiogenesis, pannus formation and erosion of bone and articular cartilage [1]. It poses a danger to both humans and animals, seriously affecting the human life quality and the healthy development of livestock and poultry farming. However, until now, there is still no safe and effective treatment for RA. Most drug treatments currently used to treat RA can only relieve clinical symptoms and delay the progression of RA to a certain extent, and all of them have certainly inevitable side effects. Therefore, it is urgent to find a much safer and more effective treatment for RA.

The nuclear factor kappa B (NF-κB) protein family is a major regulator of gene transcription involved in immune and inflammatory responses [2], which regulates the expression of proinflammatory cytokines by activating nuclear translocation of complexes in the cytoplasm [3]. Numerous studies have revealed that the activation of NF-κB p65 subunit (p65/RelA), the most important functional subunit of the NF-κB family, significantly promoted interleukin 1β (IL-1β) production in synovial fibroblasts of RA [4]. Furthermore, the p65/RelA activation is closely related to the release of proinflammatory cytokines tumor necrosis factor alpha (TNF-α) and IL-1β upon the inflammatory response [5]. Moreover, increased TNF-α and IL-1β can activate numerous signaling pathways in synovial cells. IkappaB kinase-mediated phosphorylation of IκB is able to induce the translocation of NF-κB from the cytoplasm into the nucleus and phosphorylation, which can promote nuclear transcription by binding to promoters and in turn increase the secretion of proinflammatory factors. Through this positive feedback, the NF-κB pathway is continuously activated and thereby exacerbates cellular inflammation [6].

Small interfering RNA (siRNA) is a non-coding RNA that specifically inhibits the target gene expression through RNA interference. Quinn showed that intervention with TNF-α siRNA in the early stage was able to effectively reverse immune dysregulation of RA [7]. Mesenchymal stem cells (MSCs) are multipotent stromal cells [8] that can be derived from numerous tissues of the body. MSCs and their derivatives, mainly represented by bone marrow mesenchymal stem cells (BMSCs), have been shown significant tissue repair properties by transplanting into the damaged area [9]. BMSCs can migrate to the damaged tissues and repair them with their differentiation potential. It has been proved that BMSCs can regulate B cell and T cell proliferation and exert immunosuppressive effects by releasing a soluble transforming growth factor-β1 (TGF-β1) [10]. In addition, BMSCs have a regulatory effect on immunosuppression through prostaglandin metabolites and macrophage clearance [11]. Our previous study preliminarily showed that BMSCs had good therapeutic effects on RA rats [12]. However, the effect of siRNAs targeting IL-1β/TNF-α combined with BMSCs transplantation in ameliorating RA in rats and its potential mechanism are largely unknown. Therefore, in the present study, chicken type II collagen-induced-arthritis (CIA) was employed as a RA model to comprehensively evaluate the ameliorating effect of BMSCs transplantation combined with IL-1β/TNF-α siRNAs on RA symptoms from the perspectives of growth status, behavior, immunology and pathology, and to clarify the molecular mechanism mediated by NF-κB signaling pathway, so as to provide theoretical basis for gene combined stem cell therapy for RA.

## 2. Materials and Methods

### 2.1. Animals

Eight-week-old female Wistar rats (200 ± 5 g) were gained from the Comparative Medicine Cente, Yangzhou University (certificate of quality is SYXK (Su) 2017-0007). The CIA model was induced as described previously [12]. Briefly, thirty-five rats were injected with chicken type Ⅱ collagen (cat. no. C9301; Sigma-Aldrich; Merck KGaA), twice for 4 weeks. At the beginning of CIA modeling, the first injection was performed with 1 mg/mL chicken type Ⅱ collagen by subcutaneous injection at the tail base, and the second injection was administered at the same position 1 week after the first injection. The RA symptoms were developed for totally 4 weeks. The control rats (*n* = 5) were injected with the same volume of PBS. After the second immunization, the joint lesions that were characterized by erythema and edema were daily inspected, and the severity of arthritis was monitored. Among the twenty-nine rats that developed RA symptoms, twenty-five rats were randomly selected for the subsequent experiment. All rats were housed at 23 ± 3 °C and maintained on a 12-h light/dark cycle with free access to feed and water. All procedures associated with animal care and use were approved by the Instituted Animal Care and Use Committee of Yangzhou University.

### 2.2. Experimental Design

Twenty-five CIA rats with successful modeling were randomly separated into five groups as follows: PBS (model control group, injected with 1 mL PBS), methotrexate (positive drug treatment group, injected with 1 mL 0.5 mg/mL methotrexate), BMSCs (BMSCs transplantation group, transplanted with 1 mL 1 × 10^7^/mL BMSCs), siRNA (IL-1β/TNF-α siRNAs injection group, injected with 1 mL IL-1β-siRNA and TNF-α-siRNA at a final concentration of 120 nmol/L), siRNA + BMSCs (IL-1β/TNF-α siRNAs + BMSCs transplantation group, injected separately with 1 mL IL-1β/TNF-α siRNAs and 1 mL 1 × 10^7^/mL non-transfected BMSCs), 5 rats in each group. The first injection was performed at the beginning of treatment. Seven days later, the rats were injected again with the above drugs to strengthen the therapeutic effect.

### 2.3. Design and Synthesize IL-1β/TNF-α siRNAs

IL-1β and TNF-α siRNAs were designed using siRNA design software of Invitrogen Company, and their homology was blasted in GenBank sequence database to confirm the specificity of the sequences, so as not to cause the silencing of other similar genes. The specific RNA interference fragments targeting IL-1β and TNF-α were synthesized by Invitrogen (sequences are shown in Table 1). The two siRNAs were dissolved and mixed with Entranster^TM^-R4000 (Engreen Biosystem Co., Ltd. Beijing, China). The final concentration of both siRNAs was 120 nmol/L for injection in the further therapy experiments.

### 2.4. Cell Culture and IL-1β/TNF-α siRNAs Transfection

**Figure 1:**
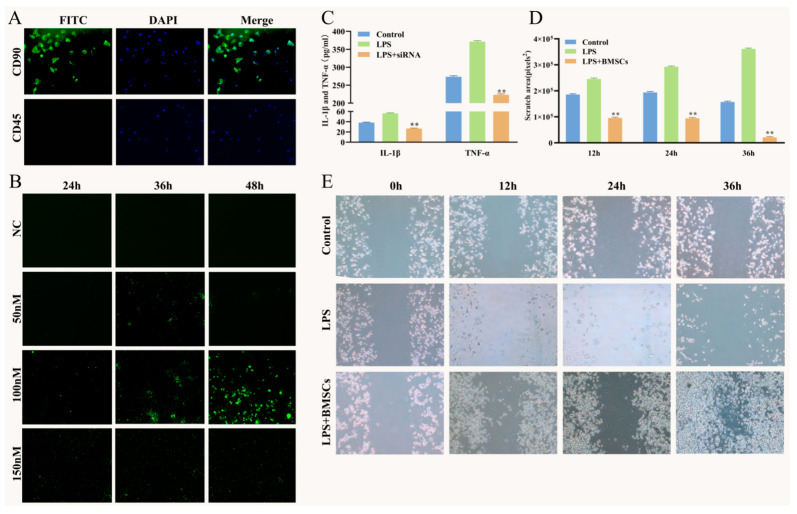
Effects of IL-1β/TNF-α and BMSCs on inflammatory factors expression and cell migration of RAW264.7. (**A**), BMSCs identification cell immunofluorescence (100 μm). (**B**), IL-1β/TNF-α siRNAs were transfected into RAW264.7 at concentrations of 50, 100 and 150 nM and observed under fluorescence microscope (200 μm). (**C**), The expression of IL-1β and TNF-α in cell supernatant of RAW264.7 by Elisa. (**D**), The scratch area of RAW264.7 cells was calculated by image (**E**), RAW264.7 cells were observed under inverted microscope at 0 h, 12 h, 24 h and 36 h after scratch experiment (100 μm). Data are presented as the mean ± SD (*n* = 6/group), ** *p* < 0.01 vs. LPS group.

BMSCs were derived from 6-week-old Wistar rats as previously described [12]. Anesthesia was performed by intraperitoneal injection of pentobarbital sodium (65 mg/kg body weight, Sigma-Aldrich, Merck KGaA. Shanghai, China) and xylazine (2 mg/kg body weight, Sigma-Aldrich, Merck KGaA. Shanghai, China), after which the rats were sacrificed for BMSCs isolation. After isolation, the BMSCs were cultured and identified, until to the third generation. CD90 and CD45 were identified by immunofluorescence of the third generation of BMSCs, as shown in Figure 1A. The BMSCs were cultured in a humidified incubator at 37 °C and 5% CO_2_. RAW264.7 were seeded in 24-well plates at an initial density of 5 × 10^4^ cells/well. When the cells reached 30–50% confluency, the transfection was respectively carried out with a final siRNA concentration of 50, 100 and 150 nM according to the instruction of EntransterTM-R4000 (Cat. No. 4000-4, Engreen Biosystem Co., Ltd. Beijing, China). They were observed under fluorescence microscope at 24, 36 and 48 h in order to determine the best transfection condition. When the density of RAW264.7 in 6-well plates reached 30–50% confluency, the transfection was carried out. After 48 h, cell supernatants and cell lysates were collected for ELISA detection. Cell scratch experiment was performed when RAW264.7 density in 6-well plate reached 50–60%. Control group was cultured with DMEM only. Lipopolysaccharide (LPS) cells were cultured with LPS (500 ng/mL in complete DMEM; cat. no. L8880, Beijing Solarbio Science & Technology Co., Ltd. Beijing, China). The LPS + BMSCs group was cultured with DMEM, 500 ng/mL LPS and BMSCs with a density of 1 × 10^5^ cells/well. They were observed under microscope at 0, 12, 24 and 36 h. 

### 2.5. In Vivo Tracing of BMSCs

The final concentration of 5-Bromo-2′-deoxyuridine (BrdU, CAS:59-14-3, Beijing Solarbio Science & Technology Co., Ltd. Beijing, China) was 10 μmol/L in α-MEM medium, which was supplemented with α-MEM basal medium (C12571500BT, Gibco, Thermo Fisher Scientific, Inc., New York, NY, USA), 10% fetal bovine serum (FBS, F8687, Sigma-Aldrich, Merck KGaA), 100 units/mL penicillin and 100 mg/mL streptomycin (15070063, Gibco, Thermo Fisher Scientific, Inc., New York, NY, USA). When the density of BMSCs reached 80% confluency, the original medium was discarded and replaced with the complete medium containing 10 μmol/L BrdU. The cells were digested after culturing for 24 h with 0.25% Trypsin-EDTA (25200072, Gibco, Thermo Fisher Scientific, Inc., New York, NY, USA) at 37 °C for 1 min. After digestion, the BMSCs were centrifuged at room temperature for 5 min at 1000 rpm. The cell pellet was resuspended in 1 mL α-MEM medium, and the cell density was adjusted to 1 × 10^7^ cells/mL.

### 2.6. Body Weight, Toe Swelling, Forced Immobility Time and Spleen Index Measurement

The body weight of each rat was recorded at the 0, 7th, 14th, 21st and 28th days after the first treatment. Meanwhile, the toe swelling of rats was measured with vernier calipers at the same position of the ankle. At the same time, the immobility time of forced swimming was measured. The day before each measurement, the rats were trained by placing them in water at 22 ± 1 °C. During the formal measurement, the rats were given 15 min’s swimming in water in advance to acclimatize to the swimming environment. Then the rats swam for another 6 min. The forced immobility time of the rats in the water was recorded when the rats stopped climbing, floated in the water and the hind toes stopped movement. In the present study, no rats were sacrificed before the completion of the experiment as a result of displaying humane endpoints such as weight loss, pain, loss of weakness or appetite.

On the 28th day after the first treatment, all rats were generally anesthetized by intraperitoneal sodium pentobarbital. Radiographs of the knee were taken with suspension type multi-function DR50 (YEMA clearXvet). Radiographs of the knee of each rat were evaluated for bone destruction on a scale of 0 = normal, 1 = mild changes, 2 = moderate changes, and 3 = severe changes [13,14]. Two observers blind to treatment assignment and with significant experience in reading and rating radiographs for patients with RA evaluated the radiographs. A total radiological score was obtained by both observers. Then the rats were sacrificed by breaking the neck, and the spleen, bilateral knee joint and surrounding synovial tissues were separated. The spleen was weighed quickly, and the spleen index that was defined as wet spleen weight/body weight (mg/g) was calculated. Spleen and right knee joint and surrounding synovial tissues were stored at −80 °C for use. The left knee joint and surrounding synovial tissue were immersed in 10% formalin.

### 2.7. Elisa

Tail vein blood sampling was performed on 14th and 28th day after the first treatment. The blood was further centrifuged at 4 °C for 5 min at 3000 rpm to obtain the serum sample. ELISA (Rat ELISA Kit Shanghai Tongwei Biological Technology Co., Ltd., Shanghai, China) was employed for detection of the serum contents of TNF-α and IL-1β according to the manufacturer’s instruction. The absorbance at 450 nm was measured.

### 2.8. Immunohistochemistry Analysis

After fixation, the knee joint and surrounding synovial tissue were decalcified with 10% EDTA (pH 7.4, CAS:60-00-4, Beijing Solarbio Science & Technology Co., Ltd., Beijing, China) for 30 days. Then the tissues were washed with running water and dehydrated, after transparent, the tissues were dipped in wax and embedded into wax blocks. The 4-μm-thick paraffin sections of joints tissue were mounted on poly-L-lysine-coated slides. Endogenous peroxidases were inhibited with 3% hydrogen peroxide. An antigen retrieval step was performed using PBS for 10 min at 92~98 °C. Unspecific binding of the antibody was prevented by 5% bovine serum albumin (BSA, CAS:9048-46-8, Beijing Solarbio Science & Technology Co., Ltd., Beijing, China) for 15 min at room temperature. The sections were then separately incubated with mouse anti-BrdU antibody (Wuhan Boster Biological Technology, Wuhan, China, 1:300 dilution), rabbit anti-NF-κB p65 antibody (AF5006, Affinity Biosciences, Suyang, China, 1: 100 dilution) and rabbit anti-phospho-NF-κB p65 antibody (AF2006, Affinity Biosciences, Changzhou, China, 1: 100 dilution) overnight. Then the sections were incubated at room temperature with a biotinylated secondary antibody for 30 min. Then the sections were incubated at 37 °C for 30 min with the streptavidin-biotin peroxidase complex (Wuhan Boster Biological Technology, Wuhan, China). The color reaction was developed by incubating the sections with diamino-benzidine (DAB, Wuhan Boster Biological Technology, Wuhan, China), and the sections were counterstained with hematoxylin. Tissue sections were viewed with inverted microscope (IX73, Olympus Corporation, Tokyo, Japan). All sections were randomized.

### 2.9. Haematoxylin & Eosin and Safranin O-Fast Green Staining

The knee joint and surrounding synovial tissue paraffin sections were deparaffinized in xylene, washed and dehydrated with a graded series of ethanol. Then sections were stained by hematoxylin and eosin (H&E) and safranin O-fast green staining (Beijing Solarbio Science & Technology Co., Ltd., Beijing, China). All sections were randomized and evaluated by two independent trained observers who were blinded to the treatment groups and the arthritis severity of each rat. Minor differences between the observers were resolved by mutual agreement. The data were expressed as inflammation, pannus, cartilage damage and bone damage scores. All scores were based on a scale of 0–3, as previously described [14,15].

### 2.10. RNA Isolation and RT-qPCR

Total RNA of spleen tissue was extracted using RNA simple Total RNA Kit (TIANGEN Biotech Co., Ltd., Beijing, China), and was reversed transcription as cDNA by FastKing gDNA Dispelling RT Supermix (TIANGEN Biotech Co., Ltd., Beijing, China) after genomic DNA was removed. Reverse transcription was performed at 42 °C for 15 min. qPCR was performed by ABI Prism 7700 sequence detection system (Applied Biosystems, Thermo Fisher Scientific, Inc., New York, NY, USA) and the reaction mix containing 1 μL cDNA, forward and reverse primers (1 µL each), 10 µL Power SYBR Green PCR Master Mix (TIANGEN Biotech Co., Ltd., Beijing, China) were fixed to a final volume of 20 µL. PCR reactions were performed as follows: initial denaturation at 95 °C for 15 min, followed by 40 cycles of denaturation at 95 °C for 10 s, annealing at 62 °C for 32 s and extension at 72 °C for 30 s, followed by a final extension at 72 °C for 10 min. β-actin was used as internal control the of mRNA expression, and the comparative 2^−ΔΔCq^ method was employed to quantify relative gene expression. The primers used in this study are listed in Table 2.

### 2.11. Statistics Analysis

Graphpad 8.0.2 was used for statistical analyses and all data were shown with the means ± standard deviation (SD). Differences between two groups and among multiple groups were evaluated by independent t-test and ordinary one-way ANOVA, respectively. *p* < 0.05 was considered statistically significant.

## 3. Results

### 3.1. IL-1β/TNF-α siRNAs and BMSCs on LPS-Induced RAW264.7 Inflammation

After isolation and culture, BMSCs were identified by immunofluorescence and results showed that CD90 was highly expressed, while CD45 has no expression (Figure 1A). Furthermore, as shown in Figure 1B, the fluorescence intensity of 50, 100 and 150 nM siRNA groups was detected at 48 h after transfection, and the 100 nM siRNA was the highest compared with the other two groups and chosen for the further experiment. Compared with LPS group, the contents of IL-1β and TNF-α in cell supernatant of RAW264.7 in LPS + siRNAs group were both significantly decreased (*p* < 0.01) after transfection with IL-1β/TNF-α siRNAs for 48 h, indicating that IL-1β/TNF-α siRNAs transfection was able to effectively inhibit the release of IL-1β and TNF-α into the cell supernatant of LPS-stimulated RAW264.7. Cell scratch results revealed that BMSCs significantly (*p* < 0.01) enhanced the migration ability of LPS-stimulated RAW264.7 (Figure 1D,E).

### 3.2. Body Weight Gain, Toe Swelling, Forced Swimming Immobility Time and Spleen Index

**Figure 2:**
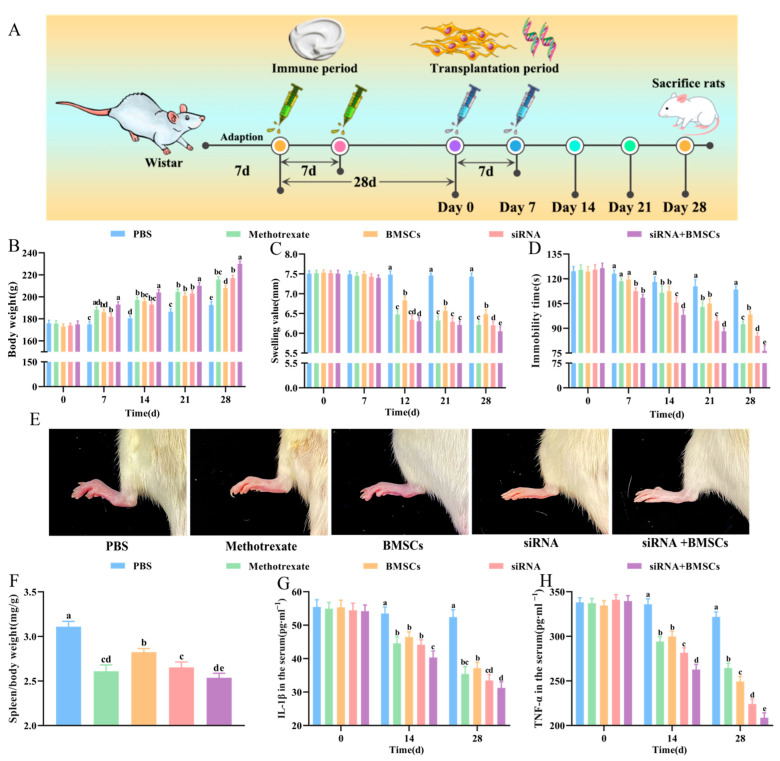
Effect of treatment with methotrexate, BMSCs, siRNA and siRNA + BMSCs on inflammation amelioration in CIA rats. (**A**), Timeline of immunization and therapy in rats. (**B**), Body weight. (**C**), Toe swelling. (**D**), The immobility time of forced swimming. (**E**), Toe swelling in CIA rats on the 28th day. (**F**), Spleen index. (**G**), Serum IL-1β concentrations. (**H**), Serum TNF-α concentrations. Values are presented as the mean ± SD, *n* = 5/group. Different letters mean significant difference among different groups (*p* < 0.05).

As shown in Figure 2B, compared with PBS rats, siRNA, BMSCs and siRNA + BMSCs rats showed significantly increased body weight and reduced toe swelling (Figure 2C). In addition, siRNA, BMSCs and siRNA + BMSCs rats presented significantly decreased immobility time of forced swimming (rats stopped struggling in water and were floating, subject to immobility of hind toes) compared with PBS rats (Figure 2D). Figure 2E showed that compared with PBS rats, the degree of joint redness was significantly ameliorated in siRNA, BMSCs and siRNA + BMSCs rats. Furthermore, our results also showed that the spleen index of the siRNA, BMSCs and siRNA + BMSCs group was significantly lower than that of the PBS group (Figure 2F), suggesting that siRNA, BMSCs and siRNA + BMSCs were all able to reduce immune intensity of CIA rats to some extent, the combination of siRNA and BMSCs has a synergistic effect on RA symptoms.

### 3.3. Serum IL-1β and TNF-α Concentrations

To further explore the effect of siRNA, BMSCs and siRNA + BMSCs on CIA rats, the spleen index and serum contents of IL-1β and TNF-α were selected for further analysis. As shown in Figure 2G,H, after treatment for 14 d and 28 d, siRNA, BMSCs and siRNA + BMSCs significantly inhibited the release of IL-1β and TNF-α into the serum compared with PBS rats, revealing that IL-1β/TNF-α siRNAs was able to play a high target gene silencing efficiency in vivo and siRNA, BMSCs and siRNA + BMSCs showed a favorable treatment effect on RA.

### 3.4. Histopathological Analysis

**Figure 3:**
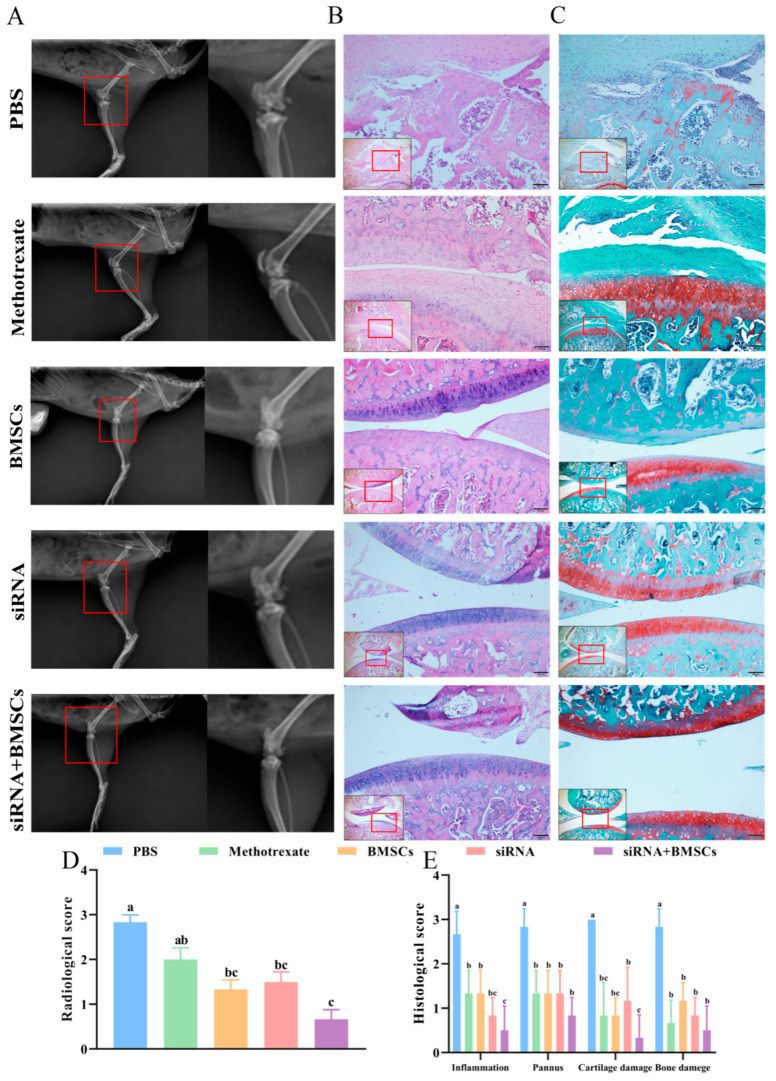
Histopathology of knee joint in CIA rats after 28 days treatment. (**A**), DR-X imaging of knee joint of CIA rats. (**B**), HE staining results of pathological slices of knee joint of CIA rats. (200 μm, 100 μm). (**C**), Safranin-O-green staining results of pathological slices of knee joint of CIA rats (200 μm, 100 μm). (**D**,**E**), The radiological and histological scores were evaluated and analyzed, respectively. Values are presented as the mean ± SD, *n* = 3/group. Different letters mean significant difference among different groups (*p* < 0.05).

As shown in Figure 3A, DR-X results showed that after 28 days of treatment, the cartilage surface edge of the knee joint was blurred, loose bodies appeared in the joint cavity and the cartilage surface was damaged with osteophyte formation in PBS rats. The siRNA + BMSCs rats had much more clear cartilage margins and significant improvement in soft tissue swelling around the knee joint. HE staining of the knee section showed that, on the 28th day after treatment, the articular cartilage lesions were characterized by erosion destruction, synovial hyperplasia and vascular pannus formation in PBS rats (Figure 3B), while the articular cartilage in the siRNA + BMSCs group was relatively smooth without vascular pannus formation and inflammatory cell infiltration. The results of safranin-O-green staining showed that the cartilage matrix was extensively absent (basically no staining) and the cartilage surface of the knee joint was severely eroded in the PBS group (Figure 3C). However, the cartilage matrix was dark red with smooth cartilage surface and arranging orderly chondrocytes in the siRNA + BMSCs group. Consistent with the clinical symptoms, DR-X results also showed that siRNA + BMSCs proved to be highly effective (Figure 3D). In addition, siRNA + BMSCs significantly reduced the scores of inflammation, pannus, cartilage damage and bone erosion compared with other groups (Figure 3E). These results indicated that the combination of BMSCs and IL-1β/TNF-α siRNAs can play a positive therapeutic effect on inflammatory relief and cartilage repair in CIA rats.

### 3.5. Immunohistochemical Analysis

**Figure 4:**
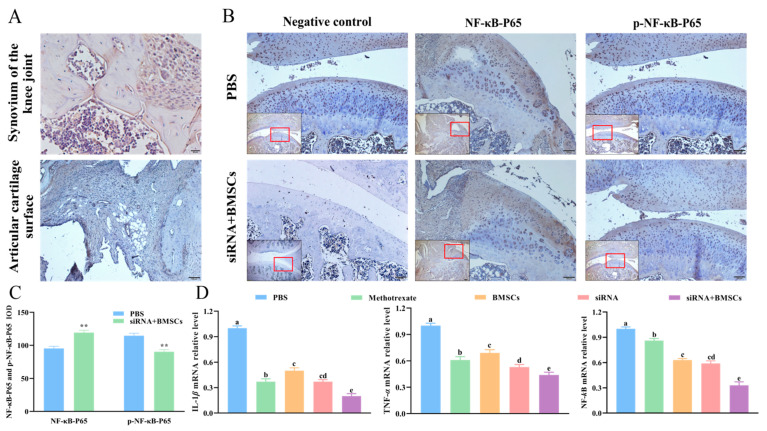
mRNA and protein expression in knee joint and spleen of CIA rats. (**A**), Observation of BMSCs labeled with BrdU under microscope (100 μm, 20 μm). (**B**), Immunohistochemical staining of NF-κB-P65 and p-NF-κB-P65 expression in joints of CIA rat’s knee (200 μm, 100 μm). (**C**), NF-κB-P65 and p-NF-κB-P65 expression. (**D**), IL-1β,TNF-α and NF-κB-P65 mRNA expression in the spleen. Data are presented as the mean ± SD (*n* = 5/group); ** *p* < 0.01 vs. PBS rats. Different letters mean significant difference (*p* < 0.05).

Results of immunohistochemical staining of knee sections showed that a large number of BrdU-labeled BMSCs could be seen in the synovium of the knee of siRNA + BMSCs rats, which were concentrated in the synovium and cartilage of the joint (Figure 4A). These results suggested that BMSCs had homing activity, and BMSCs were able to migrate and colonize the inflammatory sites and damaged joints after the tail vein injection of BMSCs. IOD results of knee joint immunohistochemistry showed that compared with PBS rats, the p-NF-κB-P65 protein level in the siRNA + BMSCs rats was significantly decreased on 28th day after the first treatment (Figure 4B,C). These results demonstrated that the combination of BMSCs and IL-1β/TNF-α siRNAs could inhibit the expression of p-NF-κB P65 and inactivate the NF-κB signaling pathway.

### 3.6. Effects of siRNA, BMSCs and siRNA + BMSCs on mRNA Expression of Inflammatory Factors in Spleen Tissue

Compared with PBS rats, the IL-1β, TNF-α and NF-κB-P65 mRNA expression in spleen tissue were significantly reduced after BMSCs, siRNA and siRNA + BMSCs treatment for 28 days. Moreover, among the three groups, siRNA + BMSCs group showed the lowest expression of IL-1β, TNF-α and NF-κB-P65 mRNA (Figure 4D), suggesting that the combination of IL-1β/TNF-α siRNAs and BMSCs was able to inhibit the IL-1β, TNF-α and NF-κB-P65 expression in vivo and BMSCs, siRNA and siRNA + BMSCs showed beneficial effect on alleviating inflammation of RA.

RA is caused by the autoimmune system dysfunction, resulting in inflammatory infiltration and erosion of articular cartilage [16]. Numerous studies showed that RA is closely related to several diseases [17,18] and has not been thoroughly cured yet, and alleviating clinical symptoms is the main treatment strategy. A survey on 2012 by McIlwraith found that about 60% of horses suffered from claudication, most of which were due to naturally occurring osteoarthritis. As the equine industry is a multi-billion dollar industry, arthritis in horses can cause millions of dollars of losses to the global economy [19]. The main clinical DMARDs used in present include methotrexate and leflunomide, which can reduce joint swelling and pain by reducing the acute marker cycoxoxidase (COX), so as to reduce joint damage and achieves anti-inflammatory and analgesic effects. Unfortunately, methotrexate has serious adverse reactions, such as pancytopenia, mucositis [20], interstitial lung disease [21] and even multiple organ failure due to poisoning [22]. Therefore, it is necessary to find a reliable and safe drug with good treatment effect for RA.

Therapies based oligonucleotide (OGN) are an emerging option that treat numerous gene-specific diseases by using nucleic acids [23]. siRNA is one of the OGN which has been widely studied in recent years. siRNAs are RNA duplexes that integrate their guide strands into the RNA-induced silencing complex in order to achieve gene-silencing effects [24]. Nusinersen is one of the siRNA agents approved by FDA for spinal muscular atrophy treatment [25]. It has been found that pro-inflammatory factors (TNF-α, IL-1 and IL-6) and chemokines (MIP-2 and IL-8) are abundant in human RA joints [26], which are the first cytokines produced by activated monocytes and macrophages during immune responses [27]. After release, these cytokines can act on non-immune system cells such as chondrocytes, osteoblasts and esendothelial cells, increasing RANKL expression and thereby increasing osteoclast production, leading to bone erosion [28]. TNF-α and IL-1β, which play an important roles in RA, appear to be involved in destructive and reparative processes [29]. TNF-α is considered to be a key mediator of chronic arthritis [30], and inhibition of TNF-α by specific antibodies has been widely welcomed as a effective treatment for RA [31]. Furthermore, TNF-α appears to be a major regulator of pro-inflammatory cytokines such as IL-1β and IL-6 [32,33]. Thus, TNF-α alone overexpression is sufficient to establish arthritis model in animals [34]. Based on the important roles of IL-1β and TNF-α in leading to RA, we designed siRNAs targeting TNF-α and IL-1β. Mononuclear macrophages are the main inflammatory cells in synovial tissues of RA patients, which can aggravate inflammation under pathological conditions. Therefore, the RAW264.7, a mouse macrophage cell line that is used extensively to carry out in vitro screens for immunomodulators, was used to establish whether siRNAs and BMSCs give the proper effects and to establish dosages in an in vitro experiment. In vitro results demonstrated that transfection with IL-1β/TNF-α siRNAs significantly reduced the contents of IL-1β and TNF-α in both cell supernatant and cell lysate in LPS-stimulated RAW264.7 compared with the control cells. These results indicated that exogenous IL-1β/TNF-α siRNAs were able to be transfected into RAW264.7 successfully and produces a highly stable silencing effect, which was consistent with our previous result [12]. Moreover, the IL-1β and TNF-α contents in the serum and the mRNA expression of IL-1β and TNF-α in spleen in IL-1β/TNF-α siRNAs rats were obviously decreased relative to PBS rats. However, the knee joints histopathology of CIA rats in the siRNA group did not show good efficacy, and this may be related to the disorder of immune cells in the joints of CIA rats under pathological conditions. Therefore, in this study, IL-1β/TNF-α siRNAs and BMSCs were combined so as to have strong ability of self-replication and multidirectional differentiation potential [35]. BMSCs are derived from bone marrow matrix, and they can regulate the T cell and B cell proliferation [36], activate immune cells to secrete soluble substances [37], such as prostaglandin E2 and IL-10 [38], and reduce inflammation responses. On the other hand, BMSCs alleviate the autoimmune response by regulating the balance between Th17/Treg [39]. BMSCs have also been shown to be effective in the treatment of osteoarthritis [40,41], degenerative arthritis [42,43] and RA in animals [12]. In particular, BMSCs has a positive effect on the articular cartilage surface repair [40]. BMSCs tracing results showed that BrdU-labeled BMSCs could proliferate at the arthritic site, and were able to be gradually induced to differentiate into osteoblasts and osteoclasts, so as to repair the damaged tissues of the joint, making BMSCs an attractive candidate for cartilage repair/regeneration therapy of on RA.

**Figure 5:**
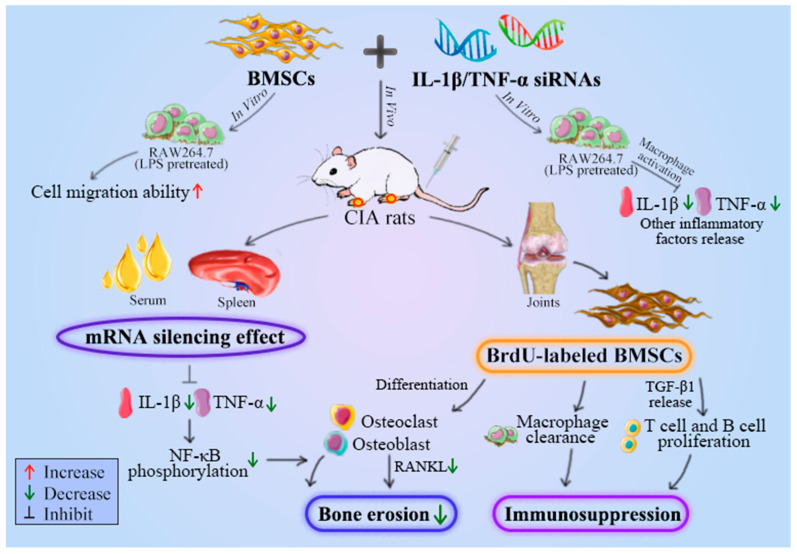
Schematic representation of proposed mechanism responsible for IL-1β/TNF-α-siRNAs combined with BMSCs mediated treatment of RA.

Therefore, in the present study, the strategy basing on siRNAs targeting both IL-1β and TNF-α combined BMSCs was used to explore the effect of CIA rats. We found that the immobility time was significantly decreased in IL-1β/TNF-α siRNA + BMSCs rats relative to PBS rats. The NF-κB-P65 in the knee joint and surrounding synovium was significantly increased, while p-NF-κB-P65 expression was significantly inhibited in IL-1β/TNF-α siRNA + BMSCs rats relative to PBS rats. Meanwhile, compared with PBS group, the IL-1β and TNF-α mRNA expression in spleen was decreased significantly in spleen of IL-1β/TNF-α siRNA + BMSCs rats, and NF-κB-P65 mRNA expression was also significantly decreased. These above results showed that tail vein injection of exogenous IL-1β/TNF-α siRNA and BMSCs showed a strong gene-silencing effect on IL-1β and TNF-α in RA rats. In addition, the immunohistochemistry results of the knee joint may be related to the promoting effect of T cells on synovitis [28], since previous study showed that T cells were able to promote synovial inflammation by promoting NF-κB-P65 activation through direct interaction with macrophages and synovial fibroblasts [44]. Importantly, cytokines-activated T cells (TH1 or TH2) were able to determine what signaling pathways (phosphoinositide 3-kinase (PI3K), NF-κB pathways, et al.) are triggered in target macrophages, and also which cytokines and chemotherapy factors are released by target macrophages [45]. In other words, the combination of IL-1β/TNF-α siRNA + BMSCs slowed down the exacerbation of inflammation, which was caused by macrophages induced NF-κB signaling pathway activation, and finally led to fewer inflammatory cell infiltrations in the knee joint and synovium and more red staining of cartilage matrix and neatly arranged chondrocytes. The fundamental mechanism of IL-1β/TNF-α-siRNAs combined with BMSCs transplantation in ameliorating rheumatoid arthritis in vivo and in vitro is summarized in Figure 5.

## 4. Conclusions

RA is a common chronic autoimmune disease resulting from inflammation and erosion of joint bone and cartilage, however, there is still a lack of effective and safe drugs to treat RA due to unknown aetiology. The exact effects and the underlying mechanisms of IL-1β/TNF-α-siRNA combined with BMSCs on RA are not well understood. In this study, the combination of BMSCs and IL-1β/TNF-α siRNAs, revealed a remarkable improvement effect on CIA rats, suggesting a synergistic effect on improving inflammation and cartilage repair in RA rats. The present study lays certain theoretical foundation for stem cell and gene therapy of RA. However, its safety and clinical efficacy still need further exploration.

## Figures and Tables

**Table 1:** siRNA sequences targeting IL-1β/TNF-α.

Target Genes	Sequences (5′-3′)
IL-1β siRNA	S: GGAAGGCAGUGUCACUCAUTT As: AUGAGUGACACUGCCUUCCTT
TNF-α siRNA	S: GGAUCUCAAAGACAACCAATT As: UUGGUUGUCUUUGAGAUCCTT

**Table 2:** qPCR primers used in the present study.

Name.	Gene Reference Number	Sequence (5′-3′)
β-actin	NM_031144.2	F: CCTCTGAACCCTAAGGCCAA R: GTCTCCGGAGTCCATCACAA
IL-1β	NM_031512.2	F: GGGATGATGACGACCTGCTA R: TGTCGTTGCTTGTCTCTCCT
TNF-α	X66539.1	F: GGTCCCAACAAGGAGGAGAA R: CTCCTCTGCTTGGTGGTTTG
NF-κB	AF079314.2	F: CGATCTGTTTCCCCTCATCTTTCC R: TGCGTCTTAGTGGTATCTGTGCTTCTC

## Data Availability

All data generated or analyzed during this study are included in this published article.

## Abbreviations

RA: rheumatoid arthritis; BMSCs: bone marrow mesenchymal stem cells; CIA: collagen-induced arthritis; IL-1β: interleukin-1β; TNF-α: tumor necrosis factor alpha; qPCR: quantitative PCR; siRNA: small interfering RNA, NF-κB: the nuclear factor kappa B.
